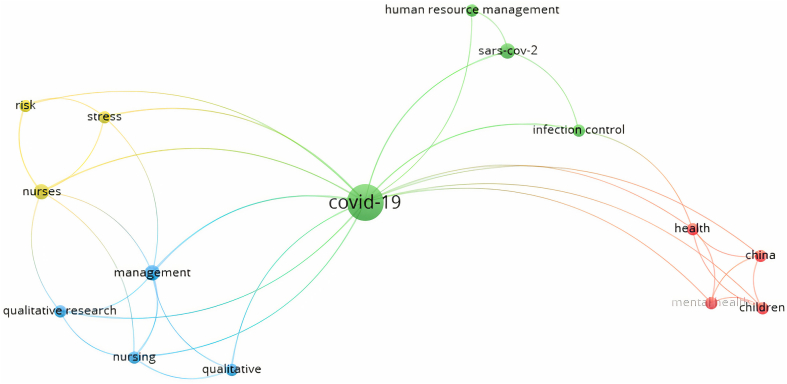# Human resource management (HRM) strategies of medical staff during the COVID-19 pandemic

**DOI:** 10.1016/j.heliyon.2023.e20355

**Published:** 2023-09-21

**Authors:** Abdullah Mahdavi, Rasha Atlasi, Maryam Ebrahimi, Ehsanollah Azimian, Roya Naemi

**Affiliations:** a Department of Health Information Management, School of Paramedical Sciences, Ardabil University of Medical Sciences, Iran; b Information and Scientometrics Center at Endocrinology and Metabolism Research Institute, Tehran University of Medical Sciences, Iran; c Department of Health Information Technology, Neyshabur University of Medical Sciences, Neyshabur, Iran; d Department of Linguistics and Foreign Languages, Payame Noor University, Tehran, Iran

**Keywords:** COVID-19, Health, HRM, Human resources management, Strategies

## Abstract

Healthcare workers are at the forefront of fight against COVID-19 and the managers of medical centers should develop coping strategies for the challenges caused by COVID-19, especially for health human resources in order to improve the performance of healthcare organizations. Hence, the purpose of this study is to investigate the human resource management strategies of medical staff during the COVID-19 to help them cope with the new strains of COVID-19 or epidemics of viral diseases that may occur in the future. In this study, a search was performed in the international Web of Science electronic database, using keywords such as human resource management and COVID-19. As a result, a total of 1884 articles published between January 1st, 2020 and October 22nd, 2021 were extracted. After screening the articles based on inclusion and exclusion criteria, 24 articles were selected to enter the study. Then, a scientometric analysis was performed on the content of selected articles and the results were presented in the form of tables and conceptual models. In total, 9 strategies were extracted from the selected articles including development of organizational culture, staff screening, policy-making, infection control training and monitoring the implementation of learned materials, patient management, human resource management, psychological and motivational support, communication and coordination, and digital health services. Employing comprehensive strategies to maintain the health of healthcare workers during the COVID-19 can play an effective role in reducing burnout, improving productivity and employee satisfaction, and in increasing the resilience of healthcare workers. It also has a positive effect on the patient's safety. Revision and reengineering of human resource management strategies in health and treatment organizations according to different cultures and contexts require research and investment in creative and innovative strategies.

## 1 Introduction

Health care organizations are one of the most complex and dynamic organizations in society [1]. Therefore, in order to achieve organizational excellence and efficiency, attention should be paid to various and extensive aspects of management such as organizational, patient, process, financial, human resources, information technology, facilities and tools, environment, and outcomes [[1], [2], [3]]. One of the issues that has attracted the attention of health care managers is human resource management (HRM) due to the lack of manpower [1].

Human resources (HRs) is considered as an important factor in the development and success of organizations in a competitive and dynamic world [4]. HRM refers to the management of people in order to improve the performance and productivity of the organization by linking HRs with organizational goals [5]. The main duties of HR managers include staffing and determining salaries, improving employees' knowledge, maintaining employees' health, changing management, adopting technology, evaluating performance, and planning [6,7]. Organizations need to identify risks and uncertainties in the organization in order to effectively manage and reduce occupational risks, motivate and retain employees, and increase productivity [7].

The World Health Organization (WHO) emphasizes on the evolving measures to resolve the emerging challenges of global health coverage under the title “*Global Human Resources Strategy for Health: Workforce 2030”* [8,9]. In the United States, the Occupational Safety and Health Administration (OSHA) require employers to provide a safe and healthy workplace for their employees [10]. Policymakers emphasize on the strong HRM policies and strategies as well as effective leadership in different situations [11]. Effective HRM, despite cultural, legal, political, and economic differences, is a major challenge for managers of health care organizations [12]. HRM in health care organizations is very complicated because staff's behavior is associated with effective and efficient care performance, patient satisfaction, patient safety, and cost reduction [13].

From 2003 to 2014, several viral epidemics have occurred in the world including the Acute Respiratory Syndrome (SARS), the influenza (caused by H1N1 virus), the Middle East Respiratory Syndrome (MERS) and the Ebola virus [14]. WHO refers to viral diseases as a serious threat to public health. A new virus was identified in early December 2019 in Wuhan, China, that was later called COVID-19. The rate of COVID-19 transmission was such that the WHO declared it an international emergency; it spread so rapidly that 81,109 laboratory-confirmed and registered cases of COVID-19 were identified in 16 days (9th to 25th of February 2020) all over the world [15].

COVID-19 has created significant challenges for the performance and sustainability of health care organizations, so managers of organizations must consciously adapt to unforeseen events in order to find strategies to deal with the possible challenges [16]. According to WHO statistics, healthcare workers are three times more likely to be infected with Covid-19 compared to the general public. They also account for 8% of all Covid-19 cases in the world. This issue is such that the people who are on the front line of fight against epidemics are called “second victims” [17,18]. Physicians and nurses are at the forefront of fight against COVID-19, which makes them be at increased risk of anxiety and stress disorders, burnout and suicide for reasons such as unpreparedness, lack of education, overwork, staff shortages, death of patients without the support of loved ones, paradoxical loyalty towards work as well as family and community commitments, and delivery of services with insufficient resources [8,19]. The short and long-term effects of COVID-19 on healthcare workers include absenteeism, migration, early retirement and job burnout [20].

Hospital managers, governments and policymakers must strive to protect healthcare workers against physical fatigue and psychological problems [21]. Communities need medical personnel to deal effectively with epidemics, so protecting them, reducing job burnout, increasing resilience and maintaining their health and well-being should be a priority [17,18,22]. During epidemics, creating a sense of security in healthcare workers and their families will improve the quality of patient care and productivity of healthcare organizations [23,24]. Saks stated that the organization's health interventions, in addition to having a positive effect on the physical and mental health, attitudes, behavior, well-being, knowledge and performance of employees, indicate that the organization cares about its HRs and this increases staff's satisfaction [25]. It is also obvious that the implementation of HRM strategies reduces the mortality rate among health care professionals, which in turn improves the patients' outcomes [26].

HR managers in health care organizations should develop and adopt targeted strategies to resolve confusion, worries and concerns of healthcare workers [27]. All countries should implement the slogan “Invest-Protect-Together” to protect their healthcare workforce [20]. The majority of studies carried out on HRM strategies of medical staff have been qualitative [[28], [29], [30]] and only few review studies have been conducted on this issue, limiting the information published in this field. Therefore, the main aim of this study is to investigate the HRM strategies implemented or proposed for medical staff during the COVID-19 pandemic with the purpose of improving the performance of healthcare organizations to deal with new and possibly dangerous strains of COVID-19 or viral diseases in the future. The results of this study can provide hospital managers, governments, and policy makers of health care organizations with the information needed to protect health care workers against occupational hazards during the epidemic. It is obvious that the use of short-term and long-term strategies to boost productivity, wellbeing, innovation, reduce job burnout, increase resilience and maintain the health of employees will create motivation and will lead to effective and efficient care and increase patient satisfaction.

## 2 Literature review

HR managers in dealing with the economic, social and uncertainty challenges of 21st century should emphasize on the employees’ training, innovative strategies, global leadership ability, flexible and cooperative strategies, and development of global thinking in order to improve effectiveness and sustainability [12]. On the other hand, for the effective management of HRs, attention should be paid to ethical and legal issues, a balance should be established between science and practice, and also the slogan of “thinking globally and acting locally” should be taken into account [6].

HR managers in hospitals should create a motivational environment to promote employee awareness and monitor employee performance to improve hospital performance in line with expected performance [31]. “Motivationally-centered leadership” was mentioned as an important HRM strategy for healthcare staff [32]. Accordingly, lack of motivation and dissatisfaction among the healthcare workers may lead to the provision of low-quality services and poor outcomes [33]. Therefore, it is recommended to create a specialized unit to provide mental health services and to give continuous financial, family and emotional support to health care professionals [34]. It has been acknowledged in a study that paying attention to internal motivational factors such as spirituality and awareness is more effective than external motivational factors such as salaries and job satisfaction of health workers [35].

Nyawira and colleagues have recommended five policies to improve HRM which include evaluating budget allocation and matching HR performance, paying health staff salaries without delay, assessing skill needs and eliminating existing gaps and optimizing skill mix, coordinating incentive conditions for staff with the same skills and roles, output-based or case-based payment for medical professionals, or a combination approach aimed at increasing efficiency [36]. In order to improve the competence and organizational commitment of medical workers, the development of HRM strategies in health care organizations requires comprehensive studies. Therefore, in addition to examining the current situation and identifying gaps, strategies can be formulated in accordance with different cultures and contexts [35,37].

Gao and colleagues showed that healthcare workers have experienced many psychological challenges during the Covid-19 pandemic including depression, anxiety, sense of helplessness, insomnia, lack of confidence in managing patients, extreme fatigue due to high workload, shortness of breath due to long-term use of personal protective equipment (PPE), and ineffective communication with patients [38]. Therefore, health care managers and policy-makers are advised to make use of appropriate self-care strategies to prevent, reduce or treat the anxiety of healthcare workers [39,40]. In a study by Philip and colleagues, it was found that distance mental health education and measures have various advantages such as cost-effectiveness, easy monitoring, and greater access to specialized care. These measures also cause positive changes in the awareness, perspective and practice of trained physicians, which in turn improve patient care [41].

During the Covid-19 pandemic, insufficient attention to employees’ empowerment, poor communication between managers and health workers, barriers to organizational culture, decision-making without a plan, and cost instead of investment were found to be among the most important challenges of HRM. Thus, the conceptual and experimental attention of scientific community is needed to deal with these challenges [4,42,43]. Policy-makers should provide a healthy work environment for healthcare workers to increase their effectiveness, productivity, innovation, accountability and commitment [44]. In a study that was conducted with the aim of improving the motivation of health workers during disasters, they identified and categorized the required interventions in five areas of leadership, education, participation, finance, and organizational policy [45].

During the Covid-19 pandemic, Azizi and colleagues examined the challenges and innovative strategies of HRM in organizations, which was well cited by the scientific community [46]. The advantages and value of these studies encouraged the authors to conduct a HRM study in a more specialized field, namely medical field. What is certain is that, the health of any country's workforce depends on the health of healthcare workers in that country. Therefore, in case of the COVID-19 pandemic continuation or emergence of new pandemics, providing occupational health for healthcare workers should be a priority for health policy-makers.

## 3 Materials and methods

### 3.1 Data source and search strategies

In this study, a search was conducted on the Web of Sciences (WOS) database, which indexes a large number of quality scientific papers. The keywords used for the search included HRM and COVID-19 (Table 1). The search strategy used in this scientometrics and literature review was developed by a clinical librarian based on the words extracted from the MeSH terms and related words. The terms were searched in the title, abstract and keywords of the articles to retrieve the most relevant articles. Also, Boolean and proximity operators were also used for a more detailed search. As a result, 1884 articles published between January 1st, 2020 and October 22nd^,^ 2021 were extracted.

**Table 1:** Search strategy used in the WOS database.

Number	Search strategy used in the WOS database
1884	Ts= ((COVID-19 OR COVID19 OR SARS-CoV-2 OR coronavirus* OR Deltacoronavirus* OR Alphacoronavirus OR Betacoronavirus OR Gammacoronavirus OR (corona AND Virus*) OR 2019-nCoV OR SARS2 OR “SARS 2″) AND ((Management* OR Administrat*) NEAR/20 (Human* OR Personnel* OR Employee* OR staff* OR Worker*)))

### 3.2 Data screening

Since the information was extracted from only one database, the step of removing duplicates was not taken in this study. Then, titles and abstracts of the extracted articles (n = 1884) were independently reviewed by R.N and M.E based on the inclusion and exclusion criteria and any disagreement was resolved and verified by A.M.

All studies that presented HRM strategies for medical or nursing staff during the COVID-19 pandemic were included in the study. Meanwhile, studies conducted on non-clinical or support workers, studies examining the impact of PPE on COVID-19, studies conducted on the nurses' experiences of mental health and its severity, review articles, letters to editor, articles related to surgery, radiology and pharmacy, and also non-English language articles were excluded from the study (n = 1,826, exclusion criteria). After screening the articles’ abstract, the full texts of 58 articles were screened carefully. From these articles, the full texts of 15 articles were not available and 19 articles were excluded from the study as they did not meet the inclusion/exclusion criteria (Fig. 1).

**Fig. 1:**
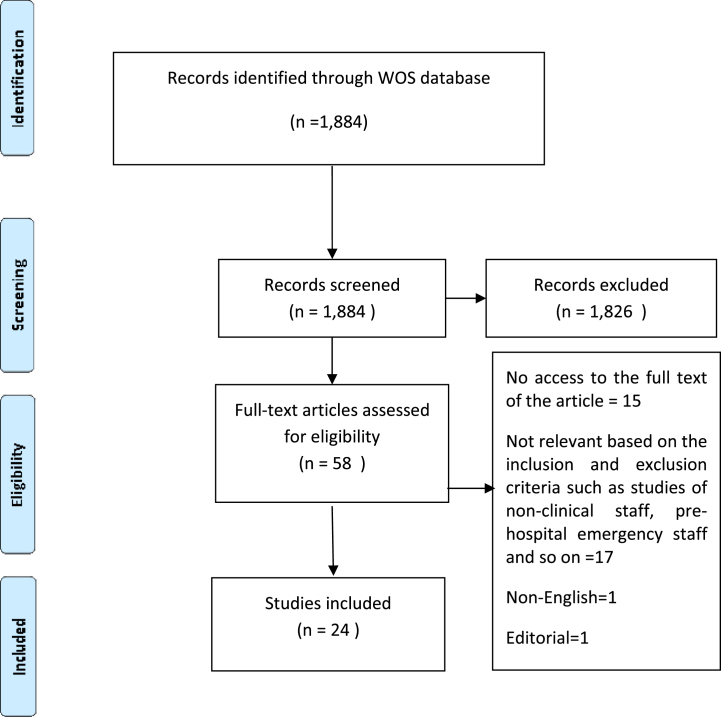
Flow diagram of searching screening and selecting process of the study.

### 3.3 Data extraction

The contents of selected articles were reviewed by two researchers (R.N and M.E) and the HRM strategies of medical staff were entered into spreadsheet. Then, in a joint meeting, the extracted strategies were discussed and the results were presented in a tabular format and the classification of the strategies was confirmed by the third researcher (A.M). Finally, the conceptual model of HRM strategies of medical staff during the COVID-19 pandemic was mapped using EdrawMax software.

### 3.4 Scientometric analysis

At the end, the scientometric analysis was performed on only 24 articles by R.A, using bibliometrix R-package and VOSviwer software. We analyzed authors, organizations, countries, journals, citations of included articles. Also, the scientific productions by countries and the co-occurrence of keywords were illustrated.

## 4 Findings

### 4.1 Scientometric analysis of the selected articles

**Fig. 2:**
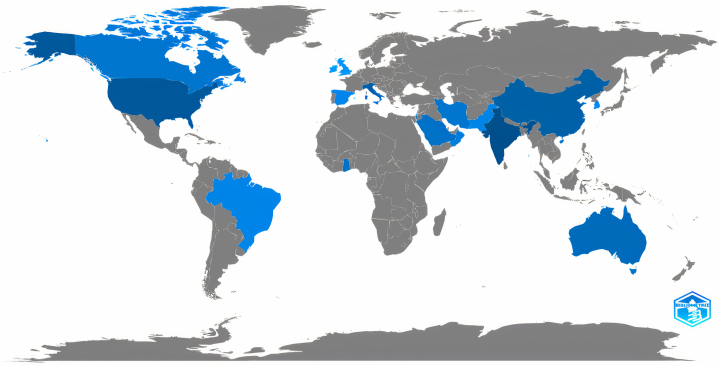
Scientific productions in the field of HRM strategies of medical staff during COVID-19 pandemic by countries.

In this study, 24 articles (published between 2019 and 2021) related to the HRM strategies for medical staff during the COVID-19 were retrieved, of which 23 were original articles (95.83%) and 1 was editorial material (4.167%). Most of the articles (14 articles, 58.33%) were published in 2021. The articles, as shown in Fig. 2, were mostly conducted with the collaboration of The United States with 5 articles (20.83%), followed by India, Italy and China each with 3 articles (12.50%) and Canada with 2 articles (8.33%), (Table 2).

**Table 2:** Number of scientific productions in the field of HRM strategies of medical staff during COVID-19 pandemic by countries.

Countries/Regions	Record Count	% of 24
USA	5	20.83%
India	3	12.50%
Italy	3	12.50%
Peoples R China	3	12.50%
Canada	2	8.33%

The retrieved articles were published in several journals, with most articles being published in the BMJ Open Journal with 3 articles (0.12%), followed by “International Journal of Environmental Research and Public Health” and “International Journal of Health Planning and Management” both with 2 articles. Most of these studies were in the field of environmental/occupational health with 8 articles (33.33%). The top 6 research areas in at least 2 articles can be seen in Table 3.

**Table 3:** Top thematic areas of related articles retrieved with at least 2 articles in that area.

Research Areas	Record Count	% of 24
Public Environmental Occupational Health	8	33.33%
General Internal Medicine	5	20.83%
Health Care Sciences Services	4	16.66%
Nursing	4	16.66%
Environmental Sciences Ecology	2	8.33%
Research Experimental Medicine	2	8.33%

**Fig. 3:**
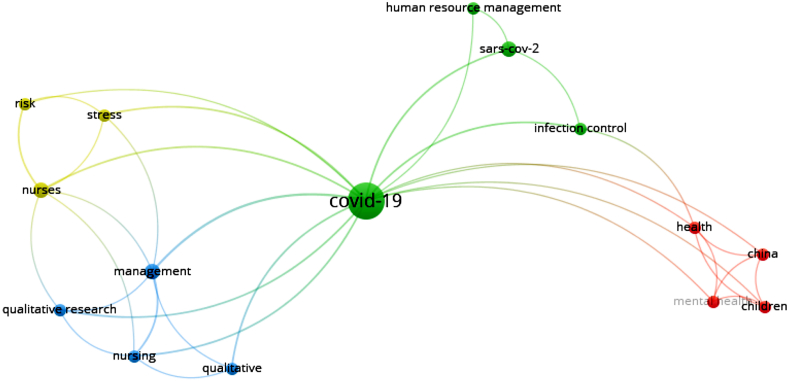
Co-occurrence network of keywords used in retrieved articles.

In the case of keywords' co-occurrence network, the words' network that occurred at least twice is presented in Fig. 3. Also, 15 out of 100 keywords used in the articles formed the largest keywords’ network, which included 4 clusters and 35 links. Following the word “COVID-19” with 16 repetitions, the words “management”, “nursing” and “mental health”, each with 3 repetitions had the most frequency. Among the retrieved articles, 5 articles with the most citations as of May 12, 2020, were identified and their information along with the information of journals that published them were determined (Table 4).

**Table 4:** Specifications of the 5 most cited articles in the field of HRM strategies of medical staff during COVID-19 pandemic.

Title	Author	Journal	Year	If	Q	Citations
Psychological Impact and Coping Strategies of Frontline Medical Staff in Hunan Between January and March 2020 During the Outbreak of Coronavirus Disease 2019 (COVID-19) in Hubei, China	Cai, HZ; et al.	Medical Science Monitor	2020	2.649	Q3	327
Robotics Utilization for Healthcare Digitization in Global COVID-19 Management	Khan, ZH; et al.	International Journal Of Environmental Research And Public Health	2020	3.39	Q1	54
Lessons from Italian front-line nurses' experiences during the COVID-19 pandemic: A qualitative descriptive study	Catania, G; et al.	Journal Of Nursing Management	2021	3.325	Q1	38
The health of healthcare professionals coping with the Covid-19 pandemic	Teixeira, CFD; et al.	Ciencia & Saude Coletiva	2020	1.336	Q4	28
Hospital staff well-being during the first wave of COVID-19: Staff perspectives	Digby, R; et al.	International Journal Of Mental Health Nursing	2021	3.503	Q1	23

### 4.2 Content analysis of selected articles

The contents of selected articles were reviewed and various strategies presented in the articles were retrieved in this study. The strategies were classified into 9 categories including the development of organizational culture, staff screening, policy-making, infection control training and monitoring the implementation of learned materials, patient management, HRM, psychological and motivational support, communication and coordination, and digital health services (Table 5).

**Table 5:** HRM strategies of medical staff during COVID-19.

Content	Strategies
Development of organizational culture	Strong governance, communication and coordination between the teams involved in responding to COVID-19 [28,75] Promoting a spirit of responsibility, adaptability, teamwork, responding to the unexpected, flexibility and overcoming resistance to change [29,30,76,77] Planning for access to more staff in an emergency to prevent disruption of medical care [78] Creating a spirit of trust and independence, promoting a spirit of decision-making, and participatory leadership [49,77,79,80] Full understanding of common priorities and goals [49] Prioritizing essential tasks and postponing unnecessary tasks [78] Adopting appropriate screening policies regarding how patients communicate with their families [78] Combining roles in examination and optimizing skill mix, sample collection or treatment, such as helping a doctor take a portable chest x-ray [30,78] Troubleshooting of work processes and rapid troubleshooting [80]
Staff screening	Not allowing the entry of employees who have fever [81,82] Performing periodic COVID-19 outbreak tests for the treatment staff [81], checking for symptoms, and timely report of temperatures above 37.3% [83] Measuring temperature and pulse oximetry at least twice a day (8:00 and 16:00; half an hour before eating, drinking or physical activity) and early detection of symptoms regardless of the workload of medical team members [28,79,81,83] Establishing COVID-19 clinic for suspected medical staff [80] Giving advice on commuting with car and avoiding the use of metro or bus [84] Prohibiting private meetings at work and maintaining a social distance of more than 1 m during emergency meetings and the use of masks [28,81,83,85] Diving strict instructions on banning any gathering during in the hospital for drinking tea or eating food, etc. [80]
Policy-making	Establishing a policy-making group on how to treat patients with COVID-19 [80] Establish three levels of management (first level: group leaders, second level: special supervisors for infection prevention and control, and third level: special head nurse or physician in each department) for daily monitoring and inspection, regular summarization and feedback, and also correction of inappropriate procedures [28,83,86] Using protective equipment with installation of reminders on doors [81] Forming crisis unit for emergency management [75] Dividing the hospital into COVID-19 and non-COVID-19 sections [80] Developing standard guidelines for preventing the spread of COVID-19 in the medical and surgical departments [80] Providing PPE for medical staff [28,75,76,78,81,83,86,87] Providing free lunches, milk, tea, snacks, mineral water, fresh fruits, juices and beverages to maintain the energy of medical staff [84,88]
Infection control training and monitoring the implementation of learned materials	Mandatory use of masks and emphasis on the correct use of PPE [78,82,84,85,87] Infection control training with the aim of increasing the knowledge of the medical staff, emphasizing the order of using PPE, the importance and how to use them [28,83,84,88] Timely training of PPE or new devices [78] Holding simulation-based workshop, for example, techniques of wearing PPE, hand hygiene, etc. [80] Teaching how to remove the PPE used when leaving COVID-19 wards, standard hand washing before and after changing clothes, changing masks, wearing outfits, disinfecting clothes with ultraviolet rays, disinfecting and wetting the soles of shoes, disinfecting the nasal cavity with iodofor or normal saline when exposed to suspicious people, disinfecting the interior of the car and washing hands frequently before eating or drinking, and before defecation [76,83,85,86] Disinfection of personal items such as glasses and mobile phones before going to work [83] Recording non-observance of health protocols, including wearing a mask, not wearing protective clothing [83] Recommending exercise at home [83] Equipping rooms with disinfecting equipment and observing rooms' cleanliness and benefiting from proper ventilation [83,88] Opening the windows at 10 a.m. and 4 p.m. for 20 min to half an hour [83] Cleaning door handles, window handles, various buttons, etc. at least twice a day [76,83,86] Providing facilities to maintain social distancing in the staff locker room and dining room by adjusting the time spent eating in different periods to reduce crowding and maintain a proper distancing (more than 1 m) when eating, and not sitting in front of each other [81,83]
Patient management	Screening of patients before hospitalization [82], patient triage [76] and telephone-based pre-triage, based on a standard questionnaire to identify COVID-19 [82] Communicating with patients infected with COVID -19 b y telephone, walkie-talkie or video program [89] Isolating patients and healthcare providers through telecommunication services [78] Limiting visits except for pediatrics, obstetrics and end-of-life patients, and assessing visitors' infectious symptoms through questionnaires and temperature screening before entering the ward [78] Hospitalizing patients with or suspected of COVID-19 in negative pressure chambers to direct airflow and remove infectious particles [78] Defining care model based on care intensity and complexity [75] Mandatory use of masks and caps for patients with COVID-19 [90] Classifying patients into urgent/emergency based on pathology, age, comorbidities, required care and availability of resources [85]
HRM	Setting 6-h shifts for nurses [86] Implementing innovative and multidisciplinary clinical care based on levels of severity and complexity [75] Dividing forces into volunteer and non-volunteer [88] Providing flexible work schedule by transferring staff to non-COVID-19 departments because of; pregnancy, lactation, treatment with immunosuppressive drugs, underlying disease [88] Continuous adjustment and transfer of nursing personnel in the ICU in terms of the number and ratio of personnel required in each shift [88] Employing new and experienced employees at the same time in each shift [30,88,91] Increasing the work shift interval for more rest and more efficient activity of the medical staff and reducing the monthly working hours by up to 30% and eliminating forced overtime [88] Managing ICU, emergency and fever clinics [80] Considering rotational shifts with the aim of preventing any bias in the assignment of tasks [80] Providing rotational leave for employees [80] Considering emergency support [80] Considering the possibility of shower and accommodation for employees [80,87] Reducing the patients -staff ratio [82] Designating nurses as coordinators [30] and assigning additional or full-time staff to lead at the national level [29]
Psychological and motivational support	Providing the possibility of rotating staff and not forcing staff to serve in COVID-19 wards [88] Providing in-person psychological preparation and psychology training in the department to reduce staff stress and anxiety at the micro and macro levels [29,30,82,87,88,92] Playing a happy morning song, performing a live concert in the hospital grounds and thanking the hospital staff [88] Surprising staff by parties, birthdays, celebrations, donating flowers and gifts to appreciate the efforts of staff [88]. Encouraging staff to serve by those with H1N1 epidemic experience [80] Offering mental health screening (anxiety and depression) with questionnaire (general anxiety) and, if necessary, providing counseling or medication [87] Using other organizational skills, for example, clergy to support mental health and mourning [29] Making sure of the awareness of all front-line personnel on the principles of emotional, physical, relational and spiritual/religious health self-care [93] Facilitating the cooperation of experienced staff with inexperienced staff to reduce stress and using the advice of senior colleagues to maintain mental health [93] Communicating with colleagues in regular team meetings with the aim of updating accurate and quality information [93] Providing separate stress management for physicians, residents, nurses [91] Crisis management at the national level with a mandatory media plan to reduce staff stress and increase staff confidence in hospitals [79] Prominent presence of veteran managers, officials and experts in morning, evening, night and holiday shifts [88]. Close communication between managers and medical staff with the aim of understanding staff fatigue and not giving remote orders [88] Distributing grants among employees working in the COVID-19 wards and equitable distribution of public, charitable aid among employees of public and private institutions [88] Timely payment of financial rewards [88] Reducing working hours and the number of shifts for more rest, and hiring temporary treatment staff [88] Installing happy messages to increase the vitality of medical staff [81] and providing social and welfare support [92]
Communication and coordination	Providing communication between medical staff and their families through video call [81] Reporting COVID-19 cases to local, provincial and national health authorities [75,80] Communicating and coordinating with government agencies such as municipalities and etc. [80] Managing the printed and electronic social media, presenting information in the form of graphs, tables and statistical analysis, and sharing information at the macro level by the Emergency Operations Command [29,80,87] Preparing and publishing posters and videos on precautionary measures and standard operating procedures at several prominent sites [80] Constantly creating and updating frequently asked questions about COVID-19 on reputable websites to prevent misconceptions in medical staff [80] Contacting the public, private and other hospitals for the provision of medical equipment [80]
Digital health services	Recommending the use of Telehealth services when necessary for medical consultation, dermatology, etc. [[80], [81], [82],^87^,^90^] Preparing and publishing training packages in WeChat group, APP platform and etc. [28,83,84,88] Providing telemedicine visits, remote monitoring and telecommunication with patients in non-emergency cases [78,80,86] Using patrol robots to measure the temperature of people, disinfection robots, hospital admission robots and nursing and telemedicine robots [64] Using electronic health records (EHR) for tracking staff vaccinations at the macro level and not setting up separate databases [29] Using information systems (website or WhatsApp group) in order to share updated information and communicate with managers to plan and reorganize the hospital [30,75,80,87].

**Fig. 4:**
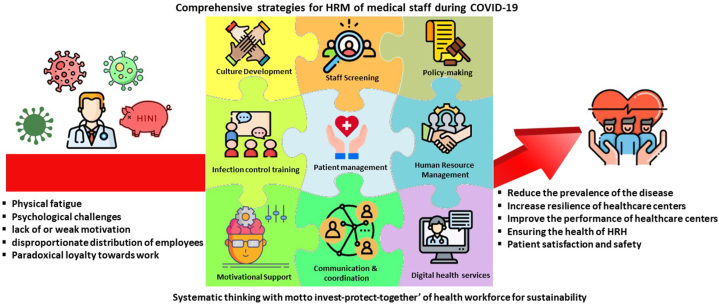
Strategies of HRM of medical staff during COVID-19 pandemic.

The main contents of strategies extracted from the articles have been presented in the form of conceptual model (Fig. 4), which can be used to maintain the health of medical staff in future crises. This model can be used by managers of healthcare organizations as a tool to analyze the current situation and determine the strengths and weaknesses of organizations. It can also be used as a road map for developing strategies to deal with future epidemics/pandemics. Therefore, the results of this study can be used to effectively manage health care HRs in order to prevent the loss of human and financial resources.

The COVID-19 pandemic has caused many problems at all levels of health systems around the world and there is a need to review and re-engineer the performance and management of health systems, especially in regard to the health HRs [8]. With the possible continuation of the COVID-19 pandemic or the emergence of new pandemics in future, provision of occupational health for healthcare workers requires systematic thinking towards enhancing the employees’ resilience and sustainability.

## 5 Discussion

Various viral pandemics/epidemics have occurred in the last 20 years, during which physicians and nurses have been at the forefront of fight against the outbreak of infectious diseases. During the Ebola epidemic, poor policies led to the escalation of disease outbreak among employees and caused a “disaster within a disaster” [18]. The Covid-19 pandemic has created complex, unstable and challenging situation for all organizations in relation to working condition and performance, as well as employees’ training, safety and health. Resolving these challenges requires innovative policies and measures that enable health care organizations to continue providing services to public and protecting their employees [47]. In this study, which was conducted with the aim of investigating the strategies used to manage and maintain the health of medical staff during the COVID-19 pandemic, 24 related articles were used, most of which were published in 2020 and the United States had the largest number of publications. The strategies extracted from these articles were classified in nine categories.

Healthcare workers in developing countries are facing challenges such as poor or lack of employees' motivation and skills, disproportionate distribution of employees, employees' low level of knowledge and lack of supervision [11,26]. Houghton and colleagues in a study found that numerous factors such as instructions on how to communicate, support of managers, organizational culture, training, access to PPE and quality of patient's care affect the ability and willingness of healthcare workers to follow the instructions set by the Unit of Control and Prevention of Respiratory Infection [48]. In the current research, the focus is on developing organizational culture as one of the nine axes of the HRM strategies of the health staff during the pandemic. This axis emphasizes strong governance, establishing communication and inter-team coordination, promoting the spirit of responsibility and collaborative leadership, flexibility, and mixed skills (Table 5).

In the meantime, participatory leadership is a tool that can strengthen the effectiveness of treatment team during COVID-19 b y relying on the activities of healthcare team members and understanding their duties and responsibilities which lead to the production of collective knowledge as well as processing and planning of advanced information [49]. Karimi Dehkordi and colleagues in their study also mentioned leadership as one of the interventions needed to increase the motivation of health workers during disasters which is in line with the findings of the present study [45]. Health care managers can enhance the employees’ motivation, mental resilience, commitment, skills, knowledge, attitude, ability and confidence by focusing on innovative HR training, psychological support, technological systems, communication and teamwork [13,38].

A comprehensive look at the motivational factors of frontline employees, the possibility of rotating employees and not forcing employees to provide services, screening employees' mental health, using clergy and experienced employees to maintain mental health, and teaching the principles of self-care for emotional, relational, and spiritual/religious health, providing financial rewards to employees working in the COVID-19 department for the use of new methods in providing medical services, are collectively effective in improving the motivation and resilience of the medical staff during the epidemic (Table 5). Establishing a specialized mental health unit and providing continuous financial, family and emotional support to health professionals is a suggestion mentioned in the study of Hameed et al. [34]. Effective management of the psychological capital of treatment staff will improve the quality of medical services, increase patient satisfaction, and bring a competitive advantage to healthcare organizations [50].

One of the challenges of the COVID-19 pandemic has been poor communication [4,42,43]. It seems that it is necessary to pay special attention to creating a stable and safe communication channel at the micro and macro level between the medical staff, government agencies and people during the epidemic in order to prevent infodemic [51] and the distribution of false information in the society. Also, creating a suitable platform for communication between the medical staff and their families during the epidemic requires serious attention. Brown-Johnson and colleagues in a qualitative study showed that factors such as providing the necessary infrastructure including employees' access to electronic health record (EHR), development of a centralized database for employees’ tracking, and use of information to update policies and protocols (such as external information sources, and reputable websites) facilitate effective communication between policymakers and health care providers [29]. McKeeby and colleagues in their study referred to asymptomatic healthcare workers as a silent threat and argued that designing and implementing an automated and efficient testing system for asymptomatic employees requires a comprehensive view of EHR, clinical information systems, and existing processes [52].

The EHR provides an opportunity for emergency patient care without the need for patient's presence [53]. Use of clinical event data in the EHR to screen healthcare workers leads to the improved epidemiological surveillance and limited contact with infected individuals, so it could be used as an appropriate and accessible strategy [54]. It seems that 0the adoption of appropriate and standard variables in order to combine information at the national and regional level and use it in the analysis and estimation of needs and informed decisions is a priority [55]. Naseri et al. have considered the use of a detailed information system including available resources, the number of graduates, staff qualifications and how to distribute HRs in the health system of countries as essential for optimal use of available resources and planning [56]. It seems that in order to compare information at the international, national and regional level, it is necessary to determine and use the standard, consistent and comprehensive data elements of HRM for health personnel. Proper management of HRs and changing organizational performance have a significant impact on improving the performance of healthcare organizations and reducing employee turnover [57].

Chandra and colleagues referred to the use of various digital health tools such as cloud computing, Virtual Reality (VR), Augmented Reality (AR), holography, three-dimensional printing, Artificial Intelligence (AI), biosensors, robots, Internet of Medical Things (IoMT), Mobile Health applications (MH), and telemedicine during the COVID-19 pandemic [58]. According to the findings of the present study, various technologies such as VR, AR, holography AI, holography, and biosensors were not mentioned by the samples of this study This indicates that healthcare workers need to be introduced to the applications of these technologies; also, they need to learn about the experience of successful countries in dealing with the pandemic through the use of these technologies (Table 5). Organizations are recommended to create new and global incentive approaches in order to apply and use appropriate strategies in emergency situations like the current pandemic [12]. Innovation and creativity are vital for sustainable and continuous development of organizations [12].

In the study of Heo and colleagues, the process of establishing telemedicine for 113 patients with COVID-19 and mild symptoms was evaluated and their satisfaction with the services provided to them was measured, showing their satisfaction score for unnecessary services to be 4.65 out of 5. The use of telemedicine services creates a positive view on the use of such services in future pandemics [59]. Tele-management of COVID-19 patients by phone with a nurse supervisor, in addition to reducing face-to-face interactions between patients and service providers, provides easy access to quality care [60]. Considering the advances made in telemedicine during the COVID-19 pandemic and also the advantages of telemedicine for patients, physicians must prepare themselves for the increasing demand for telemedicine after COVID-19 [61].

Khoshrounejad and colleagues in a study listed the causes of physicians’ resistance to telemedicine, which included high workload, low internet speed, low digital literacy, non-integration of technologies in the workflow and lack of trust in technology, lack of accurate evaluation, and lack of accessibility for the blind and deaf patients [62]. Meanwhile, Colber and colleagues referred to some of the limitations of telemedicine, which included lack of access to complete and accurate patient records, incomplete description of problems by patient, lack of quick access to diagnostic tests or imaging for accurate patient assessment, and reimbursement and billing problems [63]. The policymakers should take the necessary measures to solve the challenges related to the implementation of telemedicine. Paying attention to the use of telemedicine in developing countries and creating an index for the maturity level of societies in the field of telemedicine can be a step toward protecting HRs against future epidemics/pandemics.

Khan and colleagues showed that medical robot technology and digitalization of health care led to the increased safety and quality of health care by providing social distancing between patients and medical staff, increasing patients’ satisfaction and facilitating better clinical outcomes while reducing the workload compared to traditional and manual systems [64]. Health robots are used in various fields such as reception, nursing, telemedicine, service delivery, cleaning, surgery, radiology, screening and rehabilitation [65]. Teng and colleagues declared telepresence robots in the intensive care units as one of the most widely used robots. Telepresence is more complicated than telemedicine, as it provides the possibility of using equipment (such as a stethoscope, oximeter, otoscope, etc.) to examine patient from distance [66]. Nevertheless, issues such as high cost, political and social concern caused by the unknown consequences of using robots, limited access, inflexibility, and inability of developing countries to acquire and maintain such tools are among the challenges of using health robots [65].

Technologies based on information and communication technology (ICT), including AI, Machine Learning algorithms (ML), IoMT, big data and block-chain can be used to improve the performance and flexibility of medical robots [65]. The effective, optimal, reliable and adaptable design of medical robots require the cooperation of engineers, physicians, government and industry, and also compliance with the safety, confidentiality and clinical trial standards [67,68]. Preparedness of health care organizations to use digital health and their access to medical facilities are among vital factors that should be considered in future crises [69]. Further studies are needed to identify the challenges of digital health such as policy-making, legal and technical issues, privacy, security, reimbursement, data integration, and user-friendliness [70] with the aim of replacing and revolutionizing the current health care delivery systems [71].

Doraiswamy and colleagues suggested a global consensus to be reached in regard to the definitions of digital health, protocols such as data security and privacy, funding, comprehensive regulation, monitoring and evaluation methods [72]. The application of digital health in practice requires extensive social, educational, logistical, political, legal and organizational changes [59,73,74]. Doraiswamy and colleagues also advised developed countries to establish a coherent plan to provide health and treatment services with digital health during the current pandemic and even after that [58]. The degree of alignment of organizational strategies with the demand of HRs in the coming decades determines the success of organizations [13,29].

In the face of the Covid-19 pandemic, Zapata and colleagues proposed investing in the field of digital health and the increased recruitment of healthcare workers from countries with lower income or “source” by developing international ethics protocols [20]. In the present study, healthcare workers did not refer to the recruitment of workforce from low-income countries. So, it seems that more studies should be conducted on the positive and negative outcomes of immigration and the recruitment of healthcare workers in their country of origin and destination.

It is difficult to accurately predict the recurrence of crises such as current pandemic, so societies must be sufficiently prepared to deal with any situation in the future and protect their HRs against harm. Considering that the Covid-19 pandemic may soon be eradicated with the introduction of vaccines, and also as the health care protocols for emerging viral diseases may be different from COVID-19, the nine strategies proposed in the present study can be used as a road map of HRM of medical staff to deal with future epidemics. We should especially focus on the topics of EHR, VR, AR, AI, biosensors, robots, IoMT, MH and telemedicine, and try to overcome possible challenges and obstacles. Furthermore, in order to benefit from digital health in low and middle-income countries, feasibility studies should be conducted on the application of digital health in these countries. One of the other topics that need to be further studied is the issue of migration among healthcare workers, the reasons for migration, and positive and negative consequences of migration.

To ensure the continuity and stability of health care organizations during and after the Covid-19 pandemic, continuous studies must be conducted on the existing situation, perspectives of health care organizations in relation to global thinking and leadership approach, positive and negative consequences of digital health in HRs, and the opinions of employees and managers of health care organizations regarding the proper management of HRs in different societies. It is also suggested to pay closer attention to the following topics for future studies:

- Conducting comprehensive quantitative and qualitative studies to investigate the status and existing gaps in health care HRM in different cultures and fields with the aim of improving the professional competence and organizational commitment of healthcare workers.
- Evaluating the HRM strategies in health care organizations based on the introduced model.
- Examining the feasibility of using digital health solutions in different societies to deal with epidemic crises in the future.
- Examining the level of acceptance of digital health by managers and employees of health care organizations in dealing with the epidemics in different societies.
- Interventional study of digital health at the individual, team and organizational levels with the aim of evaluating the performance of HRs at healthcare centers.
- Conducting more studies in different societies in terms of culture, level of facilities and expectations of employees for effective planning, increasing the satisfaction of healthcare workers and enhancing the sustainability of organizations.
- Investigating the level and reasons of healthcare workers' migration and measuring the advantages of the source countries from these migrations.
- Examining the effectiveness of migrant healthcare workers in the destination countries and examining related challenges.

Conducting a comprehensive and detailed search by a clinical librarian using keywords extracted from MeSH, screening and analyzing articles extracted from a reliable database by two researchers and resolving any disagreement by a third party, and conducting a scientometric analysis on the selected articles are among the strengths of this study. The WOS database is one of the best international databases that index many high-quality scientific publications. Since various scientometric indicators (such as number of citations, indexed journals, citations to each article, etc.) are different in different databases, and as this issue created a problem in the analysis and comparison of scientometric indicators in the present study, the authors of this paper chose WOS database. The implementation of this study during the Covid-19 pandemic, the small variety of studies on this subject, and the small number of articles published during 2020–21 are among the limitations of the present study. In this study, we strived to select articles that met the inclusion/exclusion criteria by two members of the research team, but some articles may have been missed.

## 6 Conclusion

By providing comprehensive strategies for the management of healthcare workers during the COVID-19 pandemic, we can reduce the impacts of the COVID-19 pandemic, increase the resilience of medical staff and improve the performance of treatment centers. In this study, the HRM strategies used to manage healthcare workers during the Covid-19 pandemic were classified in nine strategies, including development of organizational culture, staff screening, policy-making, infection control training and monitoring the implementation of learned materials, patient management, HRM, psychological and motivational support, communication and coordination, and digital health services. Obviously, in case of the COVID-19 pandemic continuation or the emergence of new pandemics in the future, providing occupational health for health care workers should be a priority of governments in any planning in order to ensure the health of general public while maintaining the health of health care workers. Learning from this pandemic and making necessary plans for emerging diseases in the future can be effective in reducing casualties and managing health HRs, which also require research and investment in creative and innovative strategies. Healthcare organizations should use this unique situation as an opportunity to plan and invest for future pandemics. Thus, in the future, it is important to adopt and use advanced technologies such as AI, robots, and so on to deal with the challenges.

## Author contribution statement

All authors listed have significantly contributed to the development and the writing of this article.

## Data availability statement

Data will be made available on request.

## Additional information

No additional information is available for this paper.

## Declaration of competing interest

The authors declare that they have no known competing financial interests or personal relationships that could have appeared to influence the work reported in this paper.

## Figures and Tables

**figs1:**
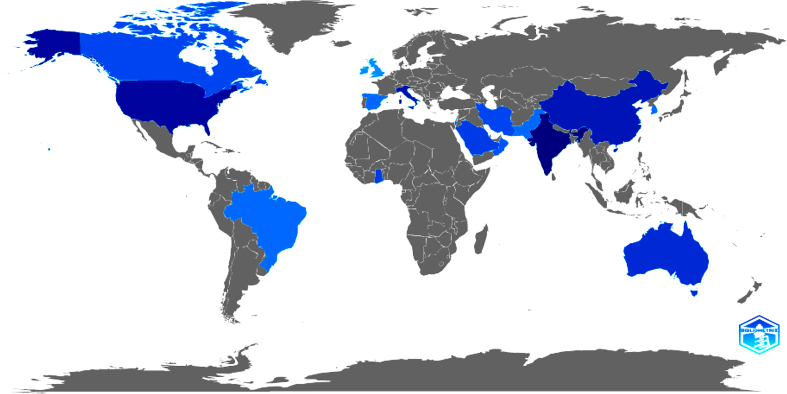


**figs2:**